# Supplementary material for: Transcriptome analysis illuminates the nature of the intracellular interaction in a vertebrate-algal symbiosis
Source: eLife. 2017 May 2;6:e22054. doi: 10.7554/eLife.22054 (PMC5413350; doi:10.7554/eLife.22054)
Supplement: Supplementary file 1. — DOI: http://dx.doi.org/10.7554/eLife.22054.028 [file elife-22054-supp1.docx]

| **Transcript ID** | **Fold change (log2)** | **Expression level (log2)** | **FDR adj. p-value** | **Uniprot ID** | **Gene Name** | **Gene Symbol** |
| --- | --- | --- | --- | --- | --- | --- |
| c4543_g1 | 7.16 | 10.72 | 1.28·10^-14^ | Q98TX3 | Programmed cell death protein 4 (Protein I11/6) | *PDCD4* |
| c477899_g2 | 5.43 | 9.81 | 1.64·10⁻⁰⁸ | Q9STT2 | Vacuolar protein sorting-associated protein 29 (Protein MAIGO 1) (Vesicle protein sorting 29) | *VPS29* |
| c300661_g1 | 5.29 | 11.51 | 1.23·10⁻⁰⁵ | P12811 | Heat shock 22 kDa protein | *HSP22* |
| c380973_g1 | 5.10 | 8.27 | 4.73·10⁻⁰⁵ | Q9LHA8 | Heat shock 70 kDa protein 4 | *MED37C* |
| c447399_g1 | 4.53 | 6.56 | 1.03·10⁻⁰⁴ | B4CUQ1 | Universal stress protein | *USP* |
| c435167_g1 | 4.43 | 9.02 | 2.98·10⁻⁰⁶ | P22242 | Desiccation-related protein PCC13-62 | *N/A* |
| c459938_g1 | 3.85 | 8.48 | 3.52·10⁻⁰⁵ | Q42684 | Superoxide dismutase [Mn], mitochondrial (EC 1.15.1.1) | *SODA* |
| c429001_g2 | 3.66 | 4.56 | 5.02·10^-03^ | Q461D5 | Stress inducible protein coi6.1 | *N/A* |
| c451611_g1 | 3.61 | 5.13 | 2.50·10^-03^ | A7E8H4 | Autophagy-related protein 8 (Autophagy-related ubiquitin-like modifier atg8) | *ATG8* |
| c448282_g1 | 3.58 | 10.58 | 1.09·10⁻⁰⁵ | Q9BYN0 | Sulfiredoxin-1 (EC 1.8.98.2) | *SRXN1* |
| c422691_g1 | 3.05 | 11.39 | 1.87·10⁻⁰⁴ | O59858 | Glutathione peroxidase (EC 1.11.1.9) | *GPX1* |
| c451611_g3 | 3.05 | 4.89 | 1.28·10^-02^ | Q9SL04 | Autophagy-related protein 8d (Autophagy-related ubiquitin-like modifier ATG8d) (AtAPG8d) (Protein autophagy 8d) | *ATG8D* |
| c395807_g1 | 2.91 | 3.24 | 1.40·10^-02^ | Q9SUR9 | Protein SGT1 homolog A (AtSGT1a) (Suppressor of G2 allele of SKP1 homolog A) | *SGT1A* |
| c473419_g1 | 2.90 | 10.31 | 3.31·10^-02^ | P12811 | Heat shock 22 kDa protein | *HSP22* |
| c904944_g1 | -2.03 | 9.16 | 4.37·10^-02^ | Q42684 | Superoxide dismutase [Mn], mitochondrial (EC 1.15.1.1) | *SODA* |
| c401130_g1 | -2.18 | 8.86 | 2.29·10^-02^ | Q10KF0 | Proteasome subunit alpha type-2 (EC 3.4.25.1) (20S proteasome alpha subunit B) (20S proteasome subunit alpha-2) | *PAB1* |
| c764117_g1 | -2.28 | 9.08 | 1.26·10^-02^ | Q8LBK6 | Monothiol glutaredoxin-S15, mitochondrial (AtGrxS15) | *GRXS15* |
| c208512_g1 | -2.54 | 9.89 | 1.54·10^-03^ | P41976 | Superoxide dismutase [Mn], mitochondrial (EC 1.15.1.1) | *SOD2* |
| c298829_g1 | -2.58 | 10.91 | 1.56·10^-03^ | P55143 | Glutaredoxin | *GLRX* |
| c369079_g1 | -2.93 | 7.85 | 5.76·10^-03^ | Q9FYK2 | Probable calcium-binding protein CML25 (Calmodulin-like protein 25) | *CML25* |
| c432897_g2 | -2.94 | 6.85 | 2.62·10^-02^ | Q9ZWQ8 | Plastid-lipid-associated protein, chloroplastic (CitPAP) | *PAP* |
| c1192053_g1 | -3.18 | 6.93 | 1.85·10^-02^ | Q9LGP4 | Os01g0120500 protein (Uncharacterized protein) | *Os01g0120500* |
| c396671_g1 | -3.19 | 8.56 | 7.63·10⁻⁰⁴ | E9S7H6 | GDSL-like protein | *GDSL-L* |
| c476229_g1 | -3.21 | 7.29 | 8.11·10^-03^ | Q8VWI1 | ER lumen protein-retaining receptor B | *ERD2B* |
| c448718_g1 | -3.39 | 6.17 | 2.21·10^-02^ | A8HPD4 | Potassium channel, NKT2-like protein | *N/A* |
| c455092_g1 | -3.50 | 6.36 | 1.31·10^-02^ | Q9FIX2 | AIG2-like protein (EC 2.3.2.-) (Putative gamma-glutamylcyclotransferase) | *NKT2L* |
| c208267_g1 | -3.54 | 6.16 | 1.85·10^-02^ | B8IHL3 | Chaperone protein DnaK (HSP70) | *AIG2L* |
| c354760_g1 | -3.90 | 5.81 | 1.46·10^-02^ | P0CW98 | Protein PLANT CADMIUM RESISTANCE 9 (AtPCR9) | *DNAK* |
| c424225_g2 | -3.92 | 5.15 | 4.04·10^-02^ | O23254 | Serine hydroxymethyltransferase 4 (AtSHMT4) (EC 2.1.2.1) (Glycine hydroxymethyltransferase 4) (Serine methylase 4) | *PCR9* |
| c452491_g1 | -4.00 | 6.02 | 1.18·10^-02^ | Q9ZUC1 | Quinone oxidoreductase-like protein At1g23740, chloroplastic (EC 1.-.-.-) | *SHM4* |
| c374044_g1 | -7.97 | 8.50 | 4.94·10⁻¹¹ | O49385 | Octicosapeptide/Phox/Bem1p and tetratricopeptide repeat domain-containing protein | *PDCD4* |

**Supplementary File 1. Differentially Expressed Stress Related Genes in *O. amblystomatis***
