# Supplementary material for: Transcriptome analysis illuminates the nature of the intracellular interaction in a vertebrate-algal symbiosis
Source: eLife. 2017 May 2;6:e22054. doi: 10.7554/eLife.22054 (PMC5413350; doi:10.7554/eLife.22054)
Supplement: Supplementary file 2. — DOI: http://dx.doi.org/10.7554/eLife.22054.029 [file elife-22054-supp2.docx]

| **Transcript ID** | **Fold change (log2)** | **Expression level (log2)** | **FDR adj. p-value** | **Uniprot ID** | **Gene Name** | **Gene Symbol** |
| --- | --- | --- | --- | --- | --- | --- |
| c464355_g1 | 6.27 | 6.91 | 3.21·10⁻⁰⁸ | B6ATE6 | Taurine catabolism dioxygenase TauD | *TAUD* |
| c448677_g2 | 4.11 | 8.71 | 3.88·10⁻⁰⁶ | A8J6J0 | Proton/sulfate cotransporter 2 | *SULTR2* |
| c419811_g2 | 3.62 | 3.82 | 2.54·10^-03^ | Q55027 | Probable chromate transport protein | *SRPC* |
| c207596_g1 | 2.94 | 7.21 | 8.68·10^-03^ | B6ATE6 | Taurine catabolism dioxygenase TauD | *TAUD* |
| c446624_g2 | 2.90 | 7.45 | 1.55·10^-02^ | O81155 | Cysteine synthase | *CS-B* |

**Supplementary File 2. Differentially Expressed Sulfur Metabolism Genes in *O. amblystomatis***
