# Supplementary material for: Transcriptome analysis illuminates the nature of the intracellular interaction in a vertebrate-algal symbiosis
Source: eLife. 2017 May 2;6:e22054. doi: 10.7554/eLife.22054 (PMC5413350; doi:10.7554/eLife.22054)
Supplement: Supplementary file 3. — DOI: http://dx.doi.org/10.7554/eLife.22054.030 [file elife-22054-supp3.docx]

| **Transcript ID** | **Fold change (log2)** | **Expression level (log2)** | **FDR adj. p-value** | **Uniprot ID** | **Gene Name** | **Gene Symbol** |
| --- | --- | --- | --- | --- | --- | --- |
| c449686_g3 | 4.70 | 6.57 | 1.98·10⁻⁰⁴ | Q46508 | NADP-reducing hydrogenase subunit HndC | *HNDD; HYDA1* |
| c377574_g1 | 4.64 | 3.92 | 1.33·10⁻⁰⁴ | Q726S7 | Phosphate acetyltransferase | *PAT* |
| c446428_g1 | 4.05 | 7.87 | 3.97·10⁻⁰⁵ | Q9S498 | 2-iminoacetate synthase | *THIH* |
| c464696_g2 | 4.03 | 8.36 | 1.33·10⁻⁰⁴ | Q8LAN3 | Probable prolyl 4-hydroxylase 4 (AtP4H4) (EC 1.14.11.2) | *P4H4* |
| c476521_g5 | 3.77 | 9.19 | 4.51·10⁻⁰⁵ | Q93092 | Transaldolase | *TALDO1* |
| c474771_g5 | 3.37 | 5.95 | 8.64·10^-03^ | Q968X7 | Pyruvate dehydrogenase | *PFOR* |
| c437767_g1 | 3.19 | 4.41 | 8.28·10^-03^ | Q9ZRF1 | Probable mannitol dehydrogenase (EC 1.1.1.255) (NAD-dependent mannitol dehydrogenase) | *CAD* |
| c400915_g1 | 3.15 | 4.71 | 6.87·10^-03^ | P9WN21 | Fructose-1,6-bisphosphatase class 2 | *GLPX* |
| c1053102_g1 | 3.05 | 7.68 | 3.17·10^-03^ | A0LG91 | Acetyl-coenzyme A synthetase | *ACSA* |
| c381246_g1 | 3.03 | 6.02 | 1.44·10^-02^ | P26563 | Aspartate aminotransferase P2 | *GOT1* |
| c473838_g1 | 3.02 | 8.66 | 1.45·10^-03^ | Q54YT4 | Trans-2-enoyl-CoA reductase, mitochondrial (EC 1.3.1.38) | *MECR* |
| c176408_g1 | 2.63 | 8.48 | 2.97·10^-02^ | P0A9Q7 | Aldehyde-alcohol dehydrogenase | *ADHE* |
| c413895_g1 | 2.44 | 12.65 | 3.42·10^-03^ | P0ACA0 | Succinate-acetate transporter protein | *SATP* |
| c481925_g1 | -2.32 | 10.25 | 7.07·10^-03^ | P55819 | Serine--glyoxylate aminotransferase | *SGAA* |
| c905937_g1 | -2.52 | 9.91 | 1.95·10^-03^ | Q7KSC4 | Mitochondrial pyruvate carrier 1 | *MPC1* |
| c1187956_g1 | -2.73 | 9.75 | 1.12·10^-03^ | Q38799 | Pyruvate dehydrogenase E1 component subunit beta-1 | *PDH2* |
| c476395_g4 | -3.29 | 5.97 | 3.52·10^-02^ | Q9SMM0 | Phosphoglucomutase | *PGMP* |

**Supplementary File 3. Differentially Expressed Genes with Roles in Fermentation in *O. amblystomatis***
