# Supplementary material for: Transcriptome analysis illuminates the nature of the intracellular interaction in a vertebrate-algal symbiosis
Source: eLife. 2017 May 2;6:e22054. doi: 10.7554/eLife.22054 (PMC5413350; doi:10.7554/eLife.22054)
Supplement: Supplementary file 4. — DOI: http://dx.doi.org/10.7554/eLife.22054.031 [file elife-22054-supp4.docx]

| **Transcript ID** | **Fold change (log2)** | **Expression level (log2)** | **FDR adj. p-value** | **Uniprot ID** | **Gene Name** | **Gene Symbol** |
| --- | --- | --- | --- | --- | --- | --- |
| c392405_g1 | 8.22 | 4.31 | 5.07·10⁻¹¹ | P22686 | Chlorophyll a-b binding protein of LHCII type I | *CAB* |
| c300443_g1 | 7.19 | 7.12 | 4.13·10⁻⁰⁹ | Q8VY88 | Protein LHCP TRANSLOCATION DEFECT | *LTD* |
| c421397_g1 | 5.78 | 7.78 | 4.21·10⁻⁰⁸ | Q9AR22 | Magnesium-protoporphyrin IX monomethyl ester [oxidative] cyclase 2 | *CTH1* |
| c457631_g1 | 4.21 | 7.71 | 1.87·10⁻⁰⁴ | Q8S091 | Thioredoxin F | *TRXF* |
| c336743_g1 | 3.72 | 4.49 | 7.02·10⁻⁰⁴ | O23920 | 4-hydroxyphenylpyruvate dioxygenase | *4HPPD* |
| c455100_g5 | 2.56 | 9.41 | 1.47·10^-02^ | P58467 | SET domain-containing protein 4 | *SETD4* |
| c325411_g1 | 2.33 | 8.65 | 3.82·10^-02^ | P35055 | Oxygen-dependent coproporphyrinogen-III oxidase | *CPX* |
| c459878_g6 | -2.04 | 9.51 | 2.62·10^-02^ | Q32RK7 | Light-independent protochlorophyllide reductase iron-sulfur ATP-binding protein | *CHLL* |
| c1188096_g1 | -2.36 | 8.90 | 1.23·10^-02^ | Q655S1 | ATP-dependent zinc metalloprotease FTSH 2 | *FTSH2* |
| c410786_g2 | -4.34 | 6.82 | 1.54·10^-03^ | P17652 | Light-independent protochlorophyllide reductase subunit B | *CHLB* |
| c764452_g1 | -7.27 | 4.51 | 2.79·10^-02^ | Q08684 | Photosystem II CP43 reaction center protein | *PSBC* |
| c455361_g2 | -7.52 | 8.12 | 1.33·10⁻⁰⁸ | P09752 | Photosystem II protein D1 | *PSBA* |
| c340838_g1 | -10.67 | 7.36 | 1.27·10⁻⁰⁷ | P10898 | Photosystem II CP43 reaction center protein | *PSBC* |

**Supplementary File 4. Differentially Expressed Genes in Photosynthesis in *O. amblystomatis***
