# Supplementary material for: Transcriptome analysis illuminates the nature of the intracellular interaction in a vertebrate-algal symbiosis
Source: eLife. 2017 May 2;6:e22054. doi: 10.7554/eLife.22054 (PMC5413350; doi:10.7554/eLife.22054)
Supplement: Supplementary file 5. — DOI: http://dx.doi.org/10.7554/eLife.22054.032 [file elife-22054-supp5.docx]

| **Transcript ID** | **Fold change (log2)** | **Expression level (log2)** | **FDR adj. p-value** | **Uniprot ID** | **Gene Name** | **Gene Symbol** |
| --- | --- | --- | --- | --- | --- | --- |
| c344647_g1 | -2.08 | 10.13 | 2.45·10^-02^ | A8IZX9 | Mitochondrial F1F0 ATP synthase associated 12.0 kDa protein | Asa9 |
| c430389_g2 | -2.10 | 10.05 | 2.29·10^-02^ | P11658 | NADH-ubiquinone oxidoreductase chain 1 (EC 1.6.5.3) (NADH dehydrogenase subunit 1) | ND1 NAD1 |
| c421949_g1 | -2.33 | 8.85 | 2.97·10^-02^ | P20113 | NADH-ubiquinone oxidoreductase chain 4 (EC 1.6.5.3) (NADH dehydrogenase subunit 4) | ND4 NAD4 |
| c396098_g1 | -2.77 | 7.58 | 2.62·10^-02^ | P08740 | NADH-ubiquinone oxidoreductase chain 2 (EC 1.6.5.3) (NADH dehydrogenase subunit 2) | ND2 NAD2 |
| c441227_g5 | -5.66 | 6.58 | 2.56·10⁻⁰⁴ | P10329 | NADH-ubiquinone oxidoreductase chain 6 (EC 1.6.5.3) (NADH dehydrogenase subunit 6) | ND6 NAD6 |
| c147070_g1 | -5.66 | 6.58 | 5.84·10⁻⁰⁵ | P23662 | Cytochrome b | MT-CYB |

**Supplementary File 5. Differentially Expressed Genes in Mitochondrial Electron Transport in *O. amblystomatis***
