# Supplementary material for: Transcriptome analysis illuminates the nature of the intracellular interaction in a vertebrate-algal symbiosis
Source: eLife. 2017 May 2;6:e22054. doi: 10.7554/eLife.22054 (PMC5413350; doi:10.7554/eLife.22054)
Supplement: Supplementary file 6. — DOI: http://dx.doi.org/10.7554/eLife.22054.033 [file elife-22054-supp6.docx]

| **Transcript ID** | **Fold change (log2)** | **Expression level (log2)** | **FDR adj. p-value** | **Uniprot ID** | **Gene Name** | **Gene Symbol** |
| --- | --- | --- | --- | --- | --- | --- |
| c424420_g2 | -2.92 | 6.24 | 4.41·10^-02^ | O82390 | Sodium-dependent phosphate transport protein 1 | *ANTR1* |
| c471617_g1 | -3.47 | 11.13 | 1.09·10⁻⁰⁵ | Q9ZPJ8 | Ammonium transporter 1 member 2 | *AMT1-2* |
| c461860_g6 | -3.88 | 7.34 | 1.54·10^-03^ | Q9FJH8 | High affinity nitrate transporter 2.4 | *NRT2.4* |
| c393951_g2 | -4.36 | 6.95 | 7.92·10⁻⁰⁴ | Q07307 | Uric acid-xanthine permease | *UAPA* |
| c477866_g1 | -4.62 | 11.72 | 1.67·10^-10^ | Q8GSD9 | Inorganic phosphate transporter 1-2 | *PTH1-2* |
| c477770_g2 | -6.00 | 7.62 | 8.38·10⁻⁰⁷ | P38361 | Phosphate permease PHO89 (Na(+)/Pi cotransporter PHO89) | *PHO89* |
| c427430_g1 | -6.68 | 10.38 | 1.28·10^-14^ | F4KD71 | High-affinity urea active transporter DUR3 | *DUR3* |

**Supplementary File 6. Differentially Expressed Nitrogen and Phosphorous Transport Genes in *O. amblystomatis***
