# Supplementary material for: Transcriptome analysis illuminates the nature of the intracellular interaction in a vertebrate-algal symbiosis
Source: eLife. 2017 May 2;6:e22054. doi: 10.7554/eLife.22054 (PMC5413350; doi:10.7554/eLife.22054)
Supplement: Supplementary file 7. — DOI: http://dx.doi.org/10.7554/eLife.22054.034 [file elife-22054-supp7.docx]

| **Transcript ID** | **Fold change (log2)** | **Expression level (log2)** | **FDR adj. p-value** | **Uniprot ID** | **Gene Name** | **PASTEClassifier-category1** | **PASTEClassifier-category2** | **PASTEClassifier-a3** | **PASTEClassifier-notes** |
| --- | --- | --- | --- | --- | --- | --- | --- | --- | --- |
| c326103_g1 | 6.66 | 0.95 | 1.18·10^-02^ | Q03275 | Retrovirus-related Pol polyprotein from type-1 retrotransposable element R2 Reverse transcriptase (EC 2.7.7.49); Endonuclease] (Fragment) | I | LINE | incomplete | CI=36; coding=(TE_BLRx: RTEX-9_ACar_2p:ClassI:LINE:RTE: 31.69%; profiles: PF13966.1_zf-RVT_NA_RT_27.5: 67.44%); struct=(TElength: >1000bps; ORF: >1000bps RT); other=(SSRCoverage=0.08) |
| c408715_g2 | 6.31 | 0.67 | 1.50·10^-02^ | Q17003 | Putative reverse transcriptase | noCat | line | NA | CI=NA; coding=(profiles: PF03372.18_Exo_endo_phos_NA_EN_19.9: 78.71%); struct=(SSRCoverage=0.10) |
| c398132_g2 | 6.12 | 0.59 | 2.76·10^-02^ |  |  | I | DIRS | incomplete | CI=16; coding=(TE_BLRx: DIRS-11B_CPB_1p:ClassI:DIRS:DIRS: 10.76%); struct=(TElength: >1000bps); other=(SSRCoverage=0.03) |
| c93734_g2 | 6.00 | 0.42 | 2.78·10^-02^ | L7MI95 | Putative tick transposon (Fragment) | I | LINE | incomplete | CI=18; coding=(TE_BLRx: RTEX-9_ACar_2p:ClassI:LINE:RTE: 21.13%; profiles: PF00078.22_RVT_1_NA_RT_20.7: 43.46%); struct=(TElength: >700bps); other=(SSRCoverage=0.07) |
| c469157_g4 | 5.87 | 0.39 | 3.26·10^-02^ | K7HPZ8 | Uncharacterized protein | I | LINE | incomplete | CI=36; coding=(TE_BLRtx: RTEX-10_ACar:ClassI:LINE:RTE: 12.41%; TE_BLRx: RTEX-10_ACar_1p:ClassI:LINE:RTE: 18.75%; profiles: PF00078.22_RVT_1_NA_RT_20.7: 61.68%); struct=(TElength: >700bps); other=(SSRCoverage=0.06) |
| c842471_g1 | 5.81 | 0.39 | 3.14·10^-02^ |  |  | I | LTR | incomplete | CI=14; coding=(TE_BLRx: ERV1-5_CPB-I_2p:ClassI:LTR:ERV: 38.82%); struct=(TElength: >700bps); other=(SSRCoverage=0.04) |
| c333341_g1 | 5.60 | 0.20 | 3.60·10^-02^ | B7S993 | Reverse transcriptase and recombinase | noCat | line | NA | CI=NA; struct=(SSRCoverage=0.05) |
| c457902_g2 | 5.51 | 0.13 | 4.28·10^-02^ | Q90Z50 | Putative reverse transcriptase | I | PLE | complete | CI=60; coding=(TE_BLRtx: Penelope-2_ACar:ClassI:PLE:Penelope: 18.82%; TE_BLRx: Penelope4_XTp:ClassI:PLE:Penelope: 31.51%); struct=(TElength: <1000bps); other=(SSRCoverage=0.05) |
| c400397_g3 | 5.50 | 0.14 | 4.25·10^-02^ | H3B4U5 | Uncharacterized protein | I | LINE | complete | CI=27; coding=(TE_BLRx: L2-2_DL_1p:ClassI:LINE:Jockey: 39.05%); struct=(TElength: >1000bps); other=(SSRCoverage=0.06) |
| c437124_g3 | 5.48 | 0.13 | 3.69·10^-02^ |  |  | I | LINE | incomplete | CI=9; coding=(profiles: PF14529.1_Exo_endo_phos_2_NA_RT_27.0: 98.32%); struct=(TElength: >700bps; polyAtail); other=(SSRCoverage=0.03) |
| c443068_g1 | 5.38 | 0.09 | 4.97·10^-02^ |  |  | I | noCat | NA | CI=33; coding=(profiles: PF13966.1_zf-RVT_NA_RT_27.5: 62.79%); other=(SSRtrf: (AAA)10_end; SSRCoverage=0.07) |
| c411939_g1 | 5.36 | 0.06 | 3.78·10^-02^ | Q7M3L4 | Putative uncharacterized protein 2 (Clone Db314) | I | LTR | incomplete | CI=7; coding=(TE_BLRx: Gypsy-2_LCh-I_1p:ClassI:LTR:Gypsy: 10.65%; profiles: _INT_gmr1_NA_INT_NA: 47.84%); struct=(TElength: <700bps); other=(SSRCoverage=0.18) |
| c456322_g9 | 5.31 | 1.21 | 2.83·10^-02^ | D7F172 | Endonuclease-reverse transcriptase | I | LINE | incomplete | CI=45; coding=(TE_BLRtx: RTEX-3_ACar:ClassI:LINE:RTE: 7.46%; TE_BLRx: RTEX-9_ACar_2p:ClassI:LINE:RTE: 28.83%; profiles: PF00078.22_RVT_1_NA_RT_20.7: 60.28%); struct=(TElength: >1000bps); other=(SSRCoverage=0.02) |
| c430811_g1 | 5.30 | 0.08 | 4.97·10^-02^ | K7JSA5 | Uncharacterized protein | II | Maverick | incomplete | CI=12; coding=(TE_BLRx: Polinton-1_SSa_5p:ClassII:Maverick:Maverick: 22.96%); struct=(TElength: <10000bps); other=(SSRCoverage=0.06) |
| c647612_g1 | 5.20 | -0.06 | 4.20·10^-02^ | K7JRQ7 | Uncharacterized protein | noCat | line | NA | CI=NA; struct=(SSRCoverage=0.13) |
| c326999_g1 | 5.15 | -0.08 | 4.46·10^-02^ | K7HPZ8 | Uncharacterized protein | noCat | line | NA | CI=NA; struct=(SSRCoverage=0.00) |
| c377233_g1 | 4.85 | 0.97 | 4.64·10^-02^ | Q03278 | Retrovirus-related Pol polyprotein from type-1 retrotransposable element R2 (Retrovirus-related Pol polyprotein from type I retrotransposable element R2) [Includes: Reverse transcriptase (EC 2.7.7.49); Endonuclease] (Fragment) | I | LINE | incomplete | CI=45; coding=(TE_BLRtx: RTEX-1_AMi:ClassI:LINE:RTE: 5.65%, RTEX-9_BF:ClassI:LINE:RTE: 14.23%; TE_BLRx: RTEX-13_ACar_2p:ClassI:LINE:RTE: 15.74%; profiles: PF00078.22_RVT_1_NA_RT_20.7: 69.63%); struct=(TElength: >1000bps); other=(SSRCoverage=0.04) |
| c479792_g3 | 4.62 | 1.21 | 3.17·10^-02^ | Q03279 | Retrovirus-related Pol polyprotein from type-1 retrotransposable element R2 (Retrovirus-related Pol polyprotein from type I retrotransposable element R2) [Includes: Reverse transcriptase (EC 2.7.7.49); Endonuclease] (Fragment) | I | LINE | incomplete | CI=45; coding=(TE_BLRtx: RTEX-10_ACar:ClassI:LINE:RTE: 12.88%, RTEX-1_ACar:ClassI:LINE:RTE: 5.71%, RTEX-20_SK:ClassI:LINE:RTE: 6.52%; TE_BLRx: RTEX-12_ACar_2p:ClassI:LINE:RTE: 23.14%, RTEX-7_SK_2p:ClassI:LINE:RTE: 11.03%; profiles: PF00078.22_RVT_1_NA_RT_20.7: 60.28%); struct=(TElength: >1000bps); other=(SSRCoverage=0.03) |
| c368651_g1 | 4.59 | 1.22 | 4.65·10^-02^ |  |  | I | LINE | complete | CI=45; coding=(TE_BLRtx: Expander1_Cis:ClassI:LINE:RTE: 8.04%, RTEX-10_ACar:ClassI:LINE:RTE: 20.93%; TE_BLRx: L1-3_Cpo_2p:ClassI:LINE:L1: 20.55%, RTEX-7_ACar_2p:ClassI:LINE:RTE: 35.61%; profiles: PF03372.18_Exo_endo_phos_NA_EN_19.9: 85.94%, PF00078.22_RVT_1_NA_RT_20.7: 93.46%); struct=(TElength: >1000bps; ORF: >1000bps); other=(SSRCoverage=0.02) |
| c377224_g1 | 4.43 | 1.44 | 4.32·10^-02^ |  |  | I | DIRS | incomplete | CI=33; coding=(TE_BLRtx: DIRS-4B_XT:ClassI:DIRS:DIRS: 6.08%; TE_BLRx: DIRS-21A_XT_3p:ClassI:DIRS:DIRS: 11.27%, DIRS-28_XT_3p:ClassI:DIRS:DIRS: 7.32%, DIRS-4_XT_2p:ClassI:DIRS:DIRS: 14.83%); struct=(TElength: >1000bps); other=(SSRCoverage=0.09) |
| c444246_g1 | 4.18 | 2.26 | 3.61·10^-02^ | Q8UWD0 | Lambda-recombinase-like protein (Fragment) | I | DIRS | incomplete | CI=33; coding=(TE_BLRtx: DIRS-23_XT:ClassI:DIRS:DIRS: 10.18%; TE_BLRx: DIRS-23_XT_2p:ClassI:DIRS:DIRS: 15.23%, DIRS-44_XT_2p:ClassI:DIRS:DIRS: 7.63%); struct=(TElength: >1000bps); other=(SSRCoverage=0.11) |
| c475054_g3 | 4.09 | 2.96 | 4.95·10⁻⁰⁴ | D7F172 | Endonuclease-reverse transcriptase | I | LINE | incomplete | CI=45; coding=(TE_BLRtx: RTEX-4_ACar:ClassI:LINE:RTE: 6.98%; TE_BLRx: RTEX-1_Crp_1p:ClassI:LINE:RTE: 31.63%; profiles: PF00078.22_RVT_1_NA_RT_20.7: 37.85%); struct=(TElength: >1000bps); other=(SSRCoverage=0.15) |
| c481105_g1 | 3.85 | 1.58 | 4.39·10^-02^ | P21328 | RNA-directed DNA polymerase from mobile element jockey (EC 2.7.7.49) (Reverse transcriptase) | I | LINE | incomplete | CI=45; coding=(TE_BLRtx: RTEX-8_ACar:ClassI:LINE:RTE: 12.09%; TE_BLRx: RTEX-11_ACar_2p:ClassI:LINE:RTE: 9.55%, RTEX-28_SK_1p:ClassI:LINE:RTE: 11.02%, RTEX-5_ACar_2p:ClassI:LINE:RTE: 10.52%, RTEX-8_ACar_2p:ClassI:LINE:RTE: 20.55%; profiles: PF00078.22_RVT_1_NA_RT_20.7: 91.59%); struct=(TElength: >1000bps); other=(SSRCoverage=0.06) |
| c462079_g1 | 1.99 | 4.18 | 3.27·10^-02^ | H3B4U5 | Uncharacterized protein | I | LINE | incomplete | CI=54; coding=(TE_BLRtx: L2-22_ACar:ClassI:LINE:Jockey: 8.57%, L2-26_CTe:ClassI:LINE:Jockey: 9.39%, L2-27_ACar:ClassI:LINE:Jockey: 6.72%, L2-2_Croc:ClassI:LINE:Jockey: 12.85%, L2-56_DR:ClassI:LINE:Jockey: 5.43%, L2-5_DRe:ClassI:LINE:Jockey: 5.12%, L2-5_GA:ClassI:LINE:Jockey: 7.99%; TE_BLRx: L2-1_CTe_1p:ClassI:LINE:Jockey: 14.39%, L2-21_ACar_2p:ClassI:LINE:Jockey: 17.24%, L2-2C_SSa_1p:ClassI:LINE:Jockey: 16.70%); struct=(TElength: >1000bps; SSRtrf: (AAACAAA)3_end, (A)24_end); other=(SSRCoverage=0.10) |
| c475521_g4 | -4.08 | 1.23 | 4.94·10^-02^ |  |  | I | LINE | complete | CI=45; coding=(TE_BLRtx: L2-2_CM:ClassI:LINE:Jockey: 5.80%, L2-6_LCh:ClassI:LINE:Jockey: 9.93%, L2-6_OL:ClassI:LINE:Jockey: 5.58%; TE_BLRx: L2-5_XT_1p:ClassI:LINE:Jockey: 35.47%); struct=(TElength: >1000bps); other=(SSRCoverage=0.06) |
| c263914_g2 | -4.24 | 1.28 | 4.92·10^-02^ |  |  | I | LINE | incomplete | CI=45; coding=(TE_BLRtx: RTEX-4_ACar:ClassI:LINE:RTE: 7.33%, RTEX-7_ACar:ClassI:LINE:RTE: 6.60%; TE_BLRx: RTE-9_NVe_2p:ClassI:LINE:RTE: 6.73%, RTEX-3_ACar_2p:ClassI:LINE:RTE: 15.26%, RTEX-6_SK_1p:ClassI:LINE:RTE: 23.59%; profiles: PF00078.22_RVT_1_NA_RT_20.7: 56.54%); struct=(TElength: >1000bps); other=(SSRCoverage=0.05) |
| c479626_g12 | -4.63 | -0.44 | 4.69·10^-02^ | P11369 | LINE-1 retrotransposable element ORF2 protein (ORF2p) (Long interspersed element-1) (L1) (Retrovirus-related Pol polyprotein LINE-1) [Includes: Reverse transcriptase (EC 2.7.7.49); Endonuclease (EC 3.1.21.-)] | I | LINE | incomplete | CI=27; coding=(TE_BLRtx: L1-67_DR:ClassI:LINE:L1: 11.62%; TE_BLRx: L1-12_DR_2p:ClassI:LINE:L1: 16.48%; profiles: PF00078.22_RVT_1_NA_RT_20.7: 23.83%); struct=(TElength: <700bps); other=(SSRCoverage=0.00) |
| c469431_g11 | -4.63 | -0.45 | 4.88·10^-02^ |  |  | I | LINE | incomplete | CI=9; coding=(TE_BLRtx: L2-6_OL:ClassI:LINE:Jockey: 5.06%); struct=(TElength: <700bps); other=(SSRCoverage=0.06) |
| c456059_g2 | -4.67 | -0.44 | 4.45·10^-02^ | O00370 | LINE-1 retrotransposable element ORF2 protein (ORF2p) [Includes: Reverse transcriptase (EC 2.7.7.49); Endonuclease (EC 3.1.21.-)] | I | LINE | incomplete | CI=27; coding=(TE_BLRtx: L1-16_Lch:ClassI:LINE:L1: 6.02%; TE_BLRx: L1-13_LCh_2p:ClassI:LINE:L1: 14.59%; profiles: PF00078.22_RVT_1_NA_RT_20.7: 40.65%); struct=(TElength: <700bps); other=(SSRCoverage=0.11) |
| c443083_g2 | -4.68 | -0.39 | 4.83·10^-02^ |  |  | I | LTR | incomplete | CI=21; coding=(TE_BLRtx: Gypsy-4_XT-I:ClassI:LTR:Gypsy: 8.85%; TE_BLRx: Gypsy95-I_DR_1p:ClassI:LTR:Gypsy: 23.51%; profiles: _RNaseH_a_clade_NA_RH_NA: 60.00%); struct=(TElength: >700bps); other=(SSRCoverage=0.00) |
| c465455_g7 | -4.70 | -0.44 | 4.71·10^-02^ | E3NSJ7 | Putative uncharacterized protein | I | LINE | incomplete | CI=27; coding=(TE_BLRtx: L2-29_ACar:ClassI:LINE:Jockey: 12.52%; TE_BLRx: L2-21_ACar_2p:ClassI:LINE:Jockey: 15.09%; profiles: PF00078.22_RVT_1_NA_RT_20.7: 61.21%); struct=(TElength: <700bps); other=(SSRCoverage=0.03) |
| c263452_g1 | -4.77 | -0.39 | 4.46·10^-02^ | F2WZX1 | Reverse transcriptase (Fragment) | I | SINE | incomplete | CI=60; struct=(TElength: <700bps; SSRtrf: (T)15_start; polyAtail); other=(TE_BLRx: L2-1_FR_1p:ClassI:LINE:Jockey: 8.69%, L2-6_XT_1p:ClassI:LINE:Jockey: 20.48%; SSRCoverage=0.23) |
| c464745_g1 | -4.80 | -0.34 | 4.64·10^-02^ |  |  | noCat | line | NA | CI=NA; struct=(SSRCoverage=0.00) |
| c377440_g1 | -4.81 | -0.34 | 4.32·10^-02^ | H2N020 | Uncharacterized protein | I | LINE | incomplete | CI=27; coding=(TE_BLRtx: L1-3_SSa:ClassI:LINE:L1: 5.64%; TE_BLRx: L1-3_SSa_2p:ClassI:LINE:L1: 12.54%); struct=(TElength: <700bps); other=(SSRCoverage=0.00) |
| c12835_g2 | -4.81 | -0.34 | 4.15·10^-02^ | Q9YGS2 | Reverse transcriptase-like protein | I | LINE | incomplete | CI=36; coding=(TE_BLRtx: CR1-L2-1_XT:ClassI:LINE:Jockey: 10.04%, L2:ClassI:LINE:Jockey: 6.23%; TE_BLRx: CR1-L2-1_XT_1p:ClassI:LINE:Jockey: 18.46%, L2-4_XT_1p:ClassI:LINE:Jockey: 20.63%); struct=(TElength: >700bps); other=(SSRCoverage=0.16) |
| c366503_g1 | -4.81 | 0.67 | 4.46·10^-02^ | H3B4U5 | Uncharacterized protein | I | LINE | incomplete | CI=36; coding=(TE_BLRtx: L2-46_DR:ClassI:LINE:Jockey: 5.36%, L2-8_OL:ClassI:LINE:Jockey: 11.78%; TE_BLRx: L2-6_OL_1p:ClassI:LINE:Jockey: 5.36%, L2-8_EL_1p:ClassI:LINE:Jockey: 15.00%, L2-8_OL_1p:ClassI:LINE:Jockey: 14.43%); struct=(TElength: >700bps); other=(SSRCoverage=0.05) |
| c633039_g1 | -4.83 | -0.34 | 4.59·10^-02^ |  |  | I | PLE | incomplete | CI=60; coding=(TE_BLRtx: Penelope-1_DR:ClassI:PLE:Penelope: 12.08%; TE_BLRx: Penelope-1_DR_2p:ClassI:PLE:Penelope: 29.09%); struct=(TElength: <1000bps); other=(SSRCoverage=0.00) |
| c329259_g1 | -4.84 | 0.86 | 3.00·10^-02^ |  |  | I | PLE | complete | CI=40; coding=(TE_BLRx: Penelope-5_ACar_1p:ClassI:PLE:Penelope: 55.02%); struct=(TElength: >1000bps); other=(SSRCoverage=0.04) |
| c19417_g1 | -4.87 | -0.34 | 3.75·10^-02^ | Q64IY1 | Reverse transcriptase (Fragment) | noCat | line | NA | CI=NA; struct=(SSRCoverage=0.04) |
| c441634_g1 | -4.89 | 0.49 | 3.17·10^-02^ | Q9YGS2 | Reverse transcriptase-like protein | I | LINE | incomplete | CI=27; coding=(TE_BLRtx: L2-2_LMe:ClassI:LINE:Jockey: 9.46%; TE_BLRx: L2-2_LMe_1p:ClassI:LINE:Jockey: 19.60%); struct=(TElength: <700bps); other=(SSRCoverage=0.09) |
| c378322_g2 | -4.91 | -0.29 | 3.78·10^-02^ | H3B4U5 | Uncharacterized protein | noCat | line | NA | CI=NA; struct=(SSRCoverage=0.29) |
| c241490_g1 | -4.93 | -0.24 | 4.15·10^-02^ | C7C202 | Gag-Pol polyprotein | I | LTR | incomplete | CI=21; coding=(TE_BLRtx: Gypsy-1_XT-I:ClassI:LTR:Gypsy: 7.92%; TE_BLRx: Gypsy-1_XT-I_1p:ClassI:LTR:Gypsy: 15.69%; profiles: _RNaseH_17_6_NA_RH_NA: 50.85%); struct=(TElength: >700bps); other=(SSRtrf: (AAAAGAAAAAA)2_end; SSRCoverage=0.09; polyAtail) |
| c436459_g1 | -4.93 | -0.25 | 4.64·10^-02^ | K7F115 | Uncharacterized protein | I | LINE | incomplete | CI=36; coding=(TE_BLRtx: L2-1_Croc:ClassI:LINE:Jockey: 9.50%, L2-2_DL:ClassI:LINE:Jockey: 23.96%; TE_BLRx: L2-1_Croc_1p:ClassI:LINE:Jockey: 15.80%, L2-20_ACar_2p:ClassI:LINE:Jockey: 16.73%; profiles: PF00078.22_RVT_1_NA_RT_20.7: 56.54%); struct=(TElength: >700bps); other=(SSRCoverage=0.02) |
| c478217_g4 | -4.95 | -0.34 | 4.32·10^-02^ | Q9YGS2 | Reverse transcriptase-like protein | I | LINE | incomplete | CI=9; coding=(TE_BLRx: L2-2_GA_1p:ClassI:LINE:Jockey: 5.41%, L2-4_XT_1p:ClassI:LINE:Jockey: 5.64%); struct=(TElength: <700bps); other=(SSRCoverage=0.24) |
| c407036_g1 | -4.96 | -0.34 | 3.88·10^-02^ | Q6AZB8 | Putative nuclease HARBI1 (EC 3.1.-.-) (Harbinger transposase-derived nuclease) | II | TIR | incomplete | CI=12; coding=(TE_BLRx: Harbinger-2_XT1p:ClassII:TIR:PIF-Harbinger: 31.83%); struct=(TElength: <700bps); other=(SSRCoverage=0.08) |
| c480935_g5 | -5.00 | -0.29 | 3.76·10^-02^ | F6Z4R1 | Uncharacterized protein | I | LINE | incomplete | CI=9; coding=(TE_BLRx: L1-18_SSa_2p:ClassI:LINE:L1: 8.65%); struct=(TElength: <700bps); other=(SSRCoverage=0.07) |
| c218406_g2 | -5.00 | -0.24 | 3.78·10^-02^ | F6KMI2 | Reverse transcriptase-like protein (Fragment) | I | LINE | incomplete | CI=27; coding=(TE_BLRtx: L2-17_CTe:ClassI:LINE:Jockey: 6.89%, L2-1_DL:ClassI:LINE:Jockey: 28.84%; TE_BLRx: L2-13B_DRe_1p:ClassI:LINE:Jockey: 10.92%, L2-1_DL_1p:ClassI:LINE:Jockey: 36.90%, L2-9_EL_1p:ClassI:LINE:Jockey: 6.90%); struct=(TElength: <700bps; SSRtrf: (TATT)7_start); other=(SSRCoverage=0.06) |
| c306252_g1 | -5.01 | -0.29 | 3.17·10^-02^ | L7MMI6 | Putative tick transposon (Fragment) | I | LTR | incomplete | CI=21; coding=(TE_BLRtx: Gypsy-8_CGi-I:ClassI:LTR:Gypsy: 7.63%; TE_BLRx: Gypsy-28_AA-I_1p:ClassI:LTR:Gypsy: 5.16%, Gypsy-87_AA-I_1p:ClassI:LTR:Gypsy: 5.38%, Gypsy-8_CGi-I_1p:ClassI:LTR:Gypsy: 10.10%); struct=(TElength: <700bps); other=(SSRCoverage=0.06) |
| c256941_g1 | -5.06 | -0.29 | 4.15·10^-02^ | P08548 | LINE-1 reverse transcriptase homolog (EC 2.7.7.49) | I | LINE | incomplete | CI=27; coding=(TE_BLRtx: L1-25_XT:ClassI:LINE:L1: 5.21%; TE_BLRx: L1-8_DR2p:ClassI:LINE:L1: 6.85%; profiles: PF00078.22_RVT_1_NA_RT_20.7: 38.79%); struct=(TElength: <700bps); other=(SSRCoverage=0.05) |
| c452666_g1 | -5.13 | -0.19 | 3.78·10^-02^ |  |  | I | LINE | incomplete | CI=9; coding=(TE_BLRx: RTEX-9_ACar_2p:ClassI:LINE:RTE: 11.17%); struct=(TElength: <700bps); other=(SSRCoverage=0.10) |
| c383954_g1 | -5.16 | -0.19 | 3.27·10^-02^ | Q17003 | Putative reverse transcriptase | I | SINE | incomplete | CI=40; struct=(TElength: <700bps; polyAtail); other=(SSRCoverage=0.17) |
| c353379_g2 | -5.22 | -0.15 | 3.17·10^-02^ | C7C202 | Gag-Pol polyprotein | I | LTR | incomplete | CI=7; coding=(TE_BLRx: Gypsy-115_AA-I_2p:ClassI:LTR:Gypsy: 6.44%, Gypsy-17_LCh-I_2p:ClassI:LTR:Gypsy: 31.05%, Gypsy13-I_SP_1p:ClassI:LTR:Gypsy: 14.77%; profiles: _RNaseH_osvaldo_NA_RH_NA: 35.25%); struct=(TElength: >700bps); other=(SSRCoverage=0.04) |
| c286327_g1 | -5.26 | -0.15 | 3.60·10^-02^ |  |  | I | LINE | incomplete | CI=36; coding=(TE_BLRtx: RTEX-3_ACar:ClassI:LINE:RTE: 7.10%, RTEX-9_ACar:ClassI:LINE:RTE: 6.33%; TE_BLRx: RTEX-9_ACar_2p:ClassI:LINE:RTE: 11.60%; profiles: PF00078.22_RVT_1_NA_RT_20.7: 54.67%); struct=(TElength: >700bps); other=(SSRCoverage=0.02) |
| c288808_g1 | -5.28 | -0.15 | 2.93·10^-02^ | H3B4U5 | Uncharacterized protein | I | LINE | incomplete | CI=9; coding=(TE_BLRx: L2-7_OL_1p:ClassI:LINE:Jockey: 19.51%); struct=(TElength: <700bps); other=(SSRCoverage=0.07) |
| c353451_g1 | -5.29 | -0.10 | 3.17·10^-02^ | E3NSJ7 | Putative uncharacterized protein | I | LINE | incomplete | CI=36; coding=(TE_BLRtx: L2:ClassI:LINE:Jockey: 14.39%; TE_BLRx: UnaL2_1p:ClassI:LINE:Jockey: 16.34%; profiles: PF00078.22_RVT_1_NA_RT_20.7: 68.69%); struct=(TElength: >700bps); other=(SSRCoverage=0.12) |
| c442452_g1 | -5.33 | 1.93 | 1.07·10^-03^ | O00370 | LINE-1 retrotransposable element ORF2 protein (ORF2p) [Includes: Reverse transcriptase (EC 2.7.7.49); Endonuclease (EC 3.1.21.-)] | I | LINE | incomplete | CI=36; coding=(TE_BLRx: L1-10_XT_2p:ClassI:LINE:L1: 5.39%, L1-1_AFC_2p:ClassI:LINE:L1: 5.66%, L1-3_LCh_2p:ClassI:LINE:L1: 12.78%); struct=(TElength: >1000bps; polyAtail); other=(SSRCoverage=0.04) |
| c219952_g1 | -5.36 | -0.02 | 3.76·10^-02^ |  |  | I | LTR | incomplete | CI=21; coding=(TE_BLRtx: Gypsy-76_AA-I:ClassI:LTR:Gypsy: 7.58%; TE_BLRx: Gypsy-158_AA-I_2p:ClassI:LTR:Gypsy: 23.02%); struct=(TElength: >700bps); other=(SSRCoverage=0.02) |
| c365681_g1 | -5.37 | -0.10 | 3.00·10^-02^ | C3YWT8 | Putative uncharacterized protein (Fragment) | I | line | NA | CI=33; coding=(profiles: PF14529.1_Exo_endo_phos_2_NA_RT_27.0: 56.30%); other=(SSRCoverage=0.09) |
| c410545_g1 | -5.40 | -0.07 | 2.96·10^-02^ | O00370 | LINE-1 retrotransposable element ORF2 protein (ORF2p) [Includes: Reverse transcriptase (EC 2.7.7.49); Endonuclease (EC 3.1.21.-)] | I | LINE | incomplete | CI=36; coding=(TE_BLRtx: L1-101_DR:ClassI:LINE:L1: 9.42%; TE_BLRx: L1-53_DR_2p:ClassI:LINE:L1: 19.15%); struct=(TElength: >700bps); other=(SSRCoverage=0.04) |
| c461441_g1 | -5.45 | -0.07 | 2.40·10^-02^ | Q90Z50 | Putative reverse transcriptase | I | PLE | complete | CI=60; coding=(TE_BLRtx: Penelope-11_XT:ClassI:PLE:Penelope: 19.24%; TE_BLRx: Penelope-11_XT_1p:ClassI:PLE:Penelope: 47.48%); struct=(TElength: <1000bps); other=(SSRCoverage=0.02) |
| c287764_g1 | -5.45 | -0.02 | 2.40·10^-02^ | Q9YGS2 | Reverse transcriptase-like protein | noCat | line | NA | CI=NA; struct=(SSRCoverage=0.11) |
| c355380_g1 | -5.45 | -0.06 | 2.31·10^-02^ | F2WZX1 | Reverse transcriptase (Fragment) | I | LINE | incomplete | CI=27; coding=(TE_BLRtx: RTEX-1_Crp:ClassI:LINE:RTE: 5.30%; TE_BLRx: RTEX-13_ACar_2p:ClassI:LINE:RTE: 17.91%, RTEX-1_Crp_1p:ClassI:LINE:RTE: 29.36%); struct=(TElength: <700bps); other=(SSRCoverage=0.07) |
| c477484_g12 | -5.71 | 0.20 | 2.76·10^-02^ | Q9YGS2 | Reverse transcriptase-like protein | I | LINE | incomplete | CI=45; coding=(TE_BLRtx: L2-2_ME:ClassI:LINE:Jockey: 5.31%, L2-6_XT:ClassI:LINE:Jockey: 9.19%; TE_BLRx: L2-6_XT_1p:ClassI:LINE:Jockey: 22.42%); struct=(TElength: >1000bps); other=(SSRCoverage=0.14) |
| c472639_g5 | -5.75 | 0.31 | 2.87·10^-02^ | E7F3G4 | Deleted. | I | LINE | incomplete | CI=27; coding=(TE_BLRx: L1-25_DR_2p:ClassI:LINE:L1: 5.95%, L1-49_DR_2p:ClassI:LINE:L1: 10.37%); struct=(TElength: >1000bps); other=(SSRCoverage=0.06) |
| c469554_g9 | -5.77 | 0.13 | 2.16·10^-02^ | B2CN80 | Reverse transcriptase | I | DIRS | incomplete | CI=25; coding=(TE_BLRtx: DIRS-8_Lch:ClassI:DIRS:DIRS: 5.66%; TE_BLRx: DIRS-8_Lch_2p:ClassI:DIRS:DIRS: 9.15%); struct=(TElength: <1000bps); other=(SSRCoverage=0.13) |
| c278549_g1 | -5.77 | 0.13 | 1.71·10^-02^ | H3B4U5 | Uncharacterized protein | noCat | line | NA | CI=NA; struct=(SSRtrf: (GTATTGTG)4_start; SSRCoverage=0.14) |
| c414402_g1 | -5.87 | 0.23 | 1.96·10^-02^ | F6Z4R1 | Uncharacterized protein | I | LINE | incomplete | CI=36; coding=(TE_BLRtx: L1-17b_Lch:ClassI:LINE:L1: 5.82%, L1-5_LCh:ClassI:LINE:L1: 6.00%, L1-7_LCh:ClassI:LINE:L1: 9.43%; TE_BLRx: L1-5_LCh_2p:ClassI:LINE:L1: 10.51%, L1-7_LCh_2p:ClassI:LINE:L1: 12.40%; profiles: PF13966.1_zf-RVT_NA_RT_27.5: 47.67%); struct=(TElength: >700bps); other=(SSRCoverage=0.07) |
| c477450_g6 | -5.91 | 0.29 | 2.44·10^-02^ | C7C202 | Gag-Pol polyprotein | I | LTR | incomplete | CI=28; coding=(TE_BLRtx: Gypsy-15_XT-I:ClassI:LTR:Gypsy: 5.81%; TE_BLRx: Gypsy-15_XT-I_2p:ClassI:LTR:Gypsy: 33.29%; profiles: PF00665.21_rve_NA_INT_30.0: 30.83%); struct=(TElength: >700bps); other=(SSRCoverage=0.04) |
| c438776_g1 | -6.48 | 0.60 | 6.70·10^-03^ | E7F3G4 |  | I | LINE | incomplete | CI=36; coding=(TE_BLRx: L1-7_SSa_2p:ClassI:LINE:L1: 7.54%); struct=(TElength: >1000bps; polyAtail); other=(SSRCoverage=0.04) |

**Supplementary File 7. Differentially Expressed Transposable Element Genes in *A. maculatum***
