# Supplementary material for: Transcriptome analysis illuminates the nature of the intracellular interaction in a vertebrate-algal symbiosis
Source: eLife. 2017 May 2;6:e22054. doi: 10.7554/eLife.22054 (PMC5413350; doi:10.7554/eLife.22054)
Supplement: Supplementary file 8. — DOI: http://dx.doi.org/10.7554/eLife.22054.035 [file elife-22054-supp8.docx]

| **Transcript ID** | **Fold change (log2)** | **Expression level (log2)** | **FDR adj. p-value** | **Uniprot ID** | **Gene Name** | **Gene Symbol** |
| --- | --- | --- | --- | --- | --- | --- |
| c400175_g1 | 6.64 | 0.92 | 1.49·10⁻⁰⁵ | Q5JTZ5 | Uncharacterized protein C9orf152 | *C9orf152* |
| c471955_g3 | 3.53 | 2.45 | 7.07·10⁻⁰⁵ | Q3ZBA3 | Pleckstrin homology domain-containing family A member 2 | *PLEKHA2* |
| c454890_g1 | 3.07 | 2.48 | 2.25·10⁻⁰⁴ | Q9P2F6 | Rho GTPase-activating protein 20 | *ARHGAP20* |
| c480474_g1 | 2.35 | 4.53 | 7.43·10⁻⁰⁶ | O93512 | GDNF family receptor alpha-4 | *GFRA4* |
| c480070_g1 | 2.06 | 3.93 | 2.51·10⁻⁰⁴ | Q9CPT0 | Apoptosis facilitator Bcl-2-like protein 14 | *BCL2L14* |
| c442057_g1 | 1.88 | 6.62 | 8.50·10⁻⁰⁵ | Q6U1I9 | Serum/glucocorticoid-regulated kinase 1 | *SGK1* |
| c429549_g1 | 1.56 | 7.17 | 7.43·10⁻⁰⁵ | Q66KM2 | Protein NDRG2 | *NDRG2* |

**Supplementary File 8. Differentially Expressed Proliferation Genes in *A. maculatum***
