# Supplementary material for: Transcriptome analysis illuminates the nature of the intracellular interaction in a vertebrate-algal symbiosis
Source: eLife. 2017 May 2;6:e22054. doi: 10.7554/eLife.22054 (PMC5413350; doi:10.7554/eLife.22054)
Supplement: Supplementary file 9. — DOI: http://dx.doi.org/10.7554/eLife.22054.036 [file elife-22054-supp9.docx]

| **Transcript ID** | **Fold change (log2)** | **Expression level (log2)** | **FDR adj. p-value** | **Uniprot ID** | **Gene Name** | **Gene Symbol** |
| --- | --- | --- | --- | --- | --- | --- |
| c444724_g1 | 9.05 | 3.21 | 6.59·10⁻⁰⁷ | P10749 | Interleukin-1 beta | *IL1B* |
| c426048_g2 | 7.40 | 1.56 | 2.01·10^-03^ | Q5KSV9 | C-X-C motif chemokine 10 | *CXCL10* |
| c332484_g1 | 6.00 | 0.63 | 3.43·10^-02^ | P10168 | Interleukin-7 | *IL7* |
| c478862_g2 | 4.58 | 4.28 | 6.18·10⁻⁰⁵ | P08317 | Interleukin-8 | *IL8* |
| c474708_g1 | 4.51 | 3.63 | 1.76·10⁻⁰⁶ | Q8CIY2 | Dual oxidase 1 | *DUOX1* |
| c456575_g1 | 3.59 | 5.63 | 1.22·10^-10^ | Q544M1 | Lymphocyte antigen 6H | *LY6H* |
| c458222_g4 | 3.29 | 4.51 | 7.89·10⁻⁰⁷ | Q2PQR2 | hepcidin | *HEP* |
| c445307_g1 | 2.89 | 5.45 | 2.22·10⁻⁰⁴ | Q90986 | Lymphocyte antigen 6E | *LY6E* |
| c80238_g1 | 2.86 | 8.10 | 1.02·10⁻⁰⁹ | Q6UX06 | Olfactomedin-4 | *OLFM4* |
| c467200_g3 | 2.05 | 5.54 | 2.01·10^-03^ | Q02496 | Mucin-1 | *MUC1* |
| c478605_g2 | 1.61 | 6.15 | 1.03·10^-02^ | Q15025 | TNFAIP3-interacting protein 1 | *TNIP1* |
| c462619_g1 | -1.45 | 5.62 | 4.88·10^-02^ | Q95JC3 | Liver-expressed antimicrobial peptide 2 | *LEAP2* |

**Supplementary File 9. Differentially Expressed Genes with Immune Functions in *A. maculatum.***
