# Supplementary material for: Transcriptome analysis illuminates the nature of the intracellular interaction in a vertebrate-algal symbiosis
Source: eLife. 2017 May 2;6:e22054. doi: 10.7554/eLife.22054 (PMC5413350; doi:10.7554/eLife.22054)
Supplement: Supplementary file 11. — DOI: http://dx.doi.org/10.7554/eLife.22054.038 [file elife-22054-supp11.docx]

| **Transcript ID** | **Fold change (log2)** | **Expression level (log2)** | **FDR adj. p-value** | **Uniprot ID** | **Gene Name** | **Gene Symbol** |
| --- | --- | --- | --- | --- | --- | --- |
| c477674_g1 | 6.33 | 3.05 | 1.80·10⁻⁰⁸ | Q687X5 | Metalloreductase STEAP4 | *STEAP4* |
| c422535_g2 | 5.55 | 0.16 | 3.27·10^-02^ | Q8N5S9 | Calcium/calmodulin-dependent protein kinase kinase 1 | *CAMKK1* |
| c451287_g2 | 4.35 | 2.28 | 3.17·10^-02^ | O15240 | N/A | *VGF* |
| c466588_g6 | 4.12 | 3.64 | 2.49·10⁻⁰⁵ | Q687X5 | Metalloreductase STEAP4 | *STEAP4* |
| c455871_g2 | 3.37 | 4.54 | 8.83·10⁻⁰⁹ | Q9JJ09 | Sodium-dependent phosphate transport protein 2B | *SLC34A2* |
| c283220_g1 | 3.16 | 4.87 | 1.69·10⁻⁰⁹ | C4N376 | Apolipoprotein L1/L2 | *APOL1/L2* |
| c474437_g1 | 2.94 | 7.03 | 2.15·10⁻⁰⁷ | Q762I5 | Resistin | *RETN* |
| c476508_g1 | 2.90 | 4.42 | 8.03·10⁻⁰⁹ | P55157 | Microsomal triglyceride transfer protein | *MTTP* |
| c460449_g3 | 2.57 | 3.30 | 2.28·10⁻⁰⁴ | Q8NBS3 | Sodium bicarbonate transporter-like protein 11 | *SLC4A11* |
| c478819_g1 | 2.46 | 5.37 | 2.47·10⁻⁰⁷ | P98164 | Low-density lipoprotein receptor-related protein 2 | *LRP2* |
| c477162_g7 | 1.92 | 6.06 | 5.11·10⁻⁰⁶ | Q9JJ09 | Sodium-dependent phosphate transport protein 2B | *SLC34A2* |
| c472618_g1 | 1.44 | 6.17 | 3.17·10^-02^ | Q9P0J1 | Pyruvate dehydrogenase phosphatase 1 | *PDP1* |
| c471279_g7 | -2.34 | 5.20 | 3.47·10⁻⁰⁶ | P24461 | Cytochrome P450 2G1 | *CYP2G1* |
| c476488_g1 | -2.56 | 5.55 | 3.06·10⁻⁰⁴ | P06727 | Apolipoprotein A-IV | *APOA4* |
| c468049_g1 | -3.07 | 6.39 | 2.87·10^-02^ | P70059 | Trypsin | *TRY* |
| c472419_g1 | -3.11 | 3.15 | 1.96·10^-02^ | O43451 | Maltase-glucoamylase | *MGAM* |
| c472419_g2 | -3.37 | 3.41 | 2.31·10^-03^ | O43451 | Maltase-glucoamylase | *MGAM* |
| c471254_g1 | -3.71 | 4.53 | 2.21·10^-02^ | Q8K2A6 | Lipase member M (EC 3.1.1.-) (Lipase-like abhydrolase domain-containing protein 3) | *LIPM* |
| c238849_g1 | -3.95 | 2.91 | 1.96·10^-02^ | P00761 | Trypsin | *TRY* |
| c418780_g1 | -3.97 | 4.02 | 2.04·10⁻¹¹ | Q7YRK6 | Cytochrome c oxidase subunit 6B1 | *COX6B1* |
| c1231732_g1 | -5.51 | -0.02 | 2.87·10^-02^ | A5D6U8 | Iron/zinc purple acid phosphatase-like protein | *PAPL* |

**Supplementary File 11. Differentially Expressed Genes in Metabolism and Nutrient Sensing in *A. maculatum.***
