# Supplementary material for: Transcriptome analysis illuminates the nature of the intracellular interaction in a vertebrate-algal symbiosis
Source: eLife. 2017 May 2;6:e22054. doi: 10.7554/eLife.22054 (PMC5413350; doi:10.7554/eLife.22054)
Supplement: Supplementary file 12. — DOI: http://dx.doi.org/10.7554/eLife.22054.039 [file elife-22054-supp12.docx]

| **Transcript ID** | **Fold change (log2)** | **Expression level (log2)** | **FDR adj. p-value** | **Uniprot ID** | **Gene Name** | **Gene Symbol** |
| --- | --- | --- | --- | --- | --- | --- |
| c449572_g1 | 4.29 | 1.95 | 4.25·10^-02^ | P51956 | Serine/threonine-protein kinase Nek3 | *NEK3* |
| c477742_g1 | 1.45 | 6.29 | 3.78·10^-02^ | P02640 | Villin-1 | *VIL1* |
| c443383_g1 | 1.40 | 8.51 | 3.17·10^-02^ | P18758 | Thymosin beta-4 | *TMSB4* |
| c451107_g1 | -1.27 | 7.75 | 4.88·10^-02^ | O42161 | Beta-actin | *ACTB* |
| c389493_g1 | -5.68 | 0.14 | 2.52·10^-02^ | Q64434 | Protein-tyrosine kinase 6 | *PTK6* |

**Supplementary File 12. Differentially Expressed Genes in Motility in *A. maculatum***
