# Supplementary material for: Transcriptome analysis illuminates the nature of the intracellular interaction in a vertebrate-algal symbiosis
Source: eLife. 2017 May 2;6:e22054. doi: 10.7554/eLife.22054 (PMC5413350; doi:10.7554/eLife.22054)
Supplement: Supplementary file 13. — DOI: http://dx.doi.org/10.7554/eLife.22054.040 [file elife-22054-supp13.docx]

| GO.ID | Term | Annotated | Significant | Expected | Rank in Elim | Fisher | Elim |
| --- | --- | --- | --- | --- | --- | --- | --- |
| GO:0015698 | inorganic anion transport | 23 | 6 | 1.07 | 1 | 0.00048 | 0.00048 |
| GO:0006085 | acetyl-CoA biosynthetic process | 5 | 3 | 0.23 | 2 | 0.00093 | 0.00093 |
| GO:0055114 | oxidation-reduction process | 489 | 40 | 22.8 | 3 | 0.00013 | 0.00165 |
| GO:0022900 | electron transport chain | 62 | 8 | 2.89 | 5 | 0.0073 | 0.0073 |
| GO:0046165 | alcohol biosynthetic process | 28 | 5 | 1.31 | 6 | 0.00846 | 0.00846 |
| GO:0019430 | removal of superoxide radicals | 10 | 3 | 0.47 | 7 | 0.00935 | 0.00935 |
| GO:0030244 | cellulose biosynthetic process | 11 | 3 | 0.51 | 10 | 0.01242 | 0.01242 |
| GO:0051274 | beta-glucan biosynthetic process | 11 | 3 | 0.51 | 11 | 0.01242 | 0.01242 |
| GO:0046686 | response to cadmium ion | 55 | 7 | 2.56 | 12 | 0.0128 | 0.0128 |
| GO:0010038 | response to metal ion | 74 | 8 | 3.45 | 17 | 0.02036 | 0.02036 |
| GO:0019751 | polyol metabolic process | 14 | 3 | 0.65 | 18 | 0.02474 | 0.02474 |
| GO:0010035 | response to inorganic substance | 137 | 14 | 6.39 | 23 | 0.004 | 0.02882 |
| GO:0022904 | respiratory electron transport chain | 27 | 4 | 1.26 | 24 | 0.03448 | 0.03448 |
| GO:0042773 | ATP synthesis coupled electron transport | 17 | 3 | 0.79 | 29 | 0.04175 | 0.04175 |
| GO:0045333 | cellular respiration | 71 | 7 | 3.31 | 30 | 0.04533 | 0.04533 |
| GO:0015994 | chlorophyll metabolic process | 57 | 6 | 2.66 | 106 | 0.04739 | 0.04739 |
| GO:0006790 | sulfur compound metabolic process | 110 | 12 | 5.13 | 107 | 0.00449 | 0.04827 |
| GO:0030243 | cellulose metabolic process | 18 | 3 | 0.84 | 108 | 0.04844 | 0.04844 |
| GO:0051273 | beta-glucan metabolic process | 18 | 3 | 0.84 | 109 | 0.04844 | 0.04844 |
| GO:0015995 | chlorophyll biosynthetic process | 44 | 5 | 2.05 | 111 | 0.05167 | 0.05167 |
| GO:0055085 | transmembrane transport | 223 | 16 | 10.4 | 112 | 0.05225 | 0.05225 |
| GO:0016125 | sterol metabolic process | 19 | 3 | 0.89 | 113 | 0.05561 | 0.05561 |
| GO:0015979 | photosynthesis | 140 | 11 | 6.53 | 114 | 0.05915 | 0.05915 |
| GO:0006914 | autophagy | 20 | 3 | 0.93 | 116 | 0.06327 | 0.06327 |
| GO:0045454 | cell redox homeostasis | 49 | 5 | 2.28 | 117 | 0.07557 | 0.07557 |

**Supplementary File 13. Top 25 Biological Process GO Annotations for Differentially Expressed *O. amblystomatis* genes.**
