# Supplementary material for: Transcriptome analysis illuminates the nature of the intracellular interaction in a vertebrate-algal symbiosis
Source: eLife. 2017 May 2;6:e22054. doi: 10.7554/eLife.22054 (PMC5413350; doi:10.7554/eLife.22054)
Supplement: Supplementary file 14. — DOI: http://dx.doi.org/10.7554/eLife.22054.041 [file elife-22054-supp14.docx]

| GO.ID | term | Annotated | Significant | Expected | Rank in Elim | Fisher | Elim |
| --- | --- | --- | --- | --- | --- | --- | --- |
| GO:0006278 | RNA-dependent DNA replication | 86 | 19 | 0.78 | 1 | 7.1E-22 | 2E-20 |
| GO:0015074 | DNA integration | 61 | 6 | 0.56 | 2 | 1.8E-05 | 2E-05 |
| GO:0090305 | nucleic acid phosphodiester bond hydrolysis | 233 | 10 | 2.12 | 3 | 4.8E-05 | 5E-05 |
| GO:0007159 | leukocyte cell-cell adhesion | 12 | 3 | 0.11 | 4 | 0.00015 | 0.0002 |
| GO:0050996 | positive regulation of lipid catabolic process | 12 | 3 | 0.11 | 5 | 0.00015 | 0.0002 |
| GO:0046683 | response to organophosphorus | 44 | 3 | 0.4 | 14 | 0.0074 | 0.0074 |
| GO:0014074 | response to purine-containing compound | 45 | 3 | 0.41 | 15 | 0.00788 | 0.0079 |
| GO:0030003 | cellular cation homeostasis | 136 | 5 | 1.24 | 16 | 0.00793 | 0.0079 |
| GO:0009617 | response to bacterium | 142 | 5 | 1.29 | 38 | 0.00946 | 0.0095 |
| GO:0055065 | metal ion homeostasis | 147 | 5 | 1.34 | 42 | 0.01089 | 0.0109 |
| GO:0043270 | positive regulation of ion transport | 55 | 3 | 0.5 | 43 | 0.01365 | 0.0137 |
| GO:0006875 | cellular metal ion homeostasis | 115 | 4 | 1.05 | 81 | 0.02071 | 0.0207 |
| GO:0045785 | positive regulation of cell adhesion | 72 | 3 | 0.66 | 110 | 0.0278 | 0.0278 |
| GO:0006869 | lipid transport | 129 | 4 | 1.18 | 112 | 0.03 | 0.03 |
| GO:0042157 | lipoprotein metabolic process | 75 | 3 | 0.68 | 115 | 0.03087 | 0.0309 |
| GO:0006814 | sodium ion transport | 83 | 3 | 0.76 | 146 | 0.0399 | 0.0399 |
| GO:0006721 | terpenoid metabolic process | 37 | 3 | 0.34 | 148 | 0.00455 | 0.0415 |
| GO:0006310 | DNA recombination | 212 | 5 | 1.93 | 150 | 0.04403 | 0.044 |
| GO:0010876 | lipid localization | 151 | 4 | 1.38 | 169 | 0.04888 | 0.0489 |
| GO:0007586 | digestion | 42 | 4 | 0.38 | 171 | 0.00055 | 0.0492 |
| GO:0006873 | cellular ion homeostasis | 143 | 6 | 1.3 | 193 | 0.0019 | 0.0594 |
| GO:0030155 | regulation of cell adhesion | 171 | 4 | 1.56 | 215 | 0.07063 | 0.0706 |
| GO:0006720 | isoprenoid metabolic process | 55 | 3 | 0.5 | 272 | 0.01365 | 0.085 |
| GO:0006955 | immune response | 427 | 8 | 3.89 | 291 | 0.04036 | 0.0889 |
| GO:0009416 | response to light stimulus | 117 | 3 | 1.07 | 292 | 0.09097 | 0.091 |

**Supplementary File 14. Top 25 Biological Process GO Annotations for Differentially Expressed *A. maculatum* genes.**
