# Supplementary material for: Transcriptome analysis illuminates the nature of the intracellular interaction in a vertebrate-algal symbiosis
Source: eLife. 2017 May 2;6:e22054. doi: 10.7554/eLife.22054 (PMC5413350; doi:10.7554/eLife.22054)
Supplement: Supplementary file 15. — DOI: http://dx.doi.org/10.7554/eLife.22054.042 [file elife-22054-supp15.docx]

| term_ID | description | frequency | log10 p-value | uniqueness | dispensability |
| --- | --- | --- | --- | --- | --- |
| GO:0015698 | inorganic anion transport | 0.77% | -3.3188 | 0.866 | 0 |
| GO:0006637 | acyl-CoA metabolic process | 0.22% | -2.7773 | 0.833 | 0 |
| GO:0010035 | response to inorganic substance | 0.27% | -2.3979 | 0.843 | 0 |
| GO:0044699 | single-organism process | 53.98% | -1.6031 | 0.997 | 0 |
| GO:0042592 | homeostatic process | 0.97% | -1.0696 | 0.908 | 0 |
| GO:0044706 | multi-multicellular organism process | 0.02% | -1.051 | 0.956 | 0 |
| GO:0008152 | metabolic process | 82.18% | -0.5058 | 0.999 | 0 |
| GO:0050896 | response to stimulus | 8.82% | -0.452 | 0.995 | 0 |
| GO:0023052 | signaling | 3.84% | -0.3163 | 0.994 | 0 |
| GO:0051704 | multi-organism process | 2.77% | -0.2478 | 0.994 | 0 |
| GO:0051179 | localization | 17.86% | -0.2381 | 0.995 | 0 |
| GO:0022414 | reproductive process | 0.12% | -0.1764 | 0.994 | 0 |
| GO:0002376 | immune system process | 0.63% | -0.1627 | 0.994 | 0 |
| GO:0000003 | reproduction | 0.21% | -0.1277 | 1 | 0 |
| GO:0065007 | biological regulation | 14.92% | -0.0226 | 0.995 | 0 |
| GO:0032259 | methylation | 2.78% | -0.294 | 0.984 | 0.007 |
| GO:0009056 | catabolic process | 9.63% | -0.1499 | 0.982 | 0.012 |
| GO:0006914 | autophagy | 0.10% | -1.1988 | 0.898 | 0.044 |
| GO:0072593 | reactive oxygen species metabolic process | 0.20% | -0.7372 | 0.943 | 0.046 |
| GO:1901615 | organic hydroxy compound metabolic process | 0.99% | -1.1379 | 0.945 | 0.047 |
| GO:0008283 | cell proliferation | 0.11% | -0.4394 | 0.954 | 0.048 |
| GO:0006915 | apoptotic process | 0.25% | -0.5145 | 0.901 | 0.052 |
| GO:0016265 | death | 0.28% | -0.5029 | 0.95 | 0.053 |
| GO:0006790 | sulfur compound metabolic process | 1.67% | -2.3478 | 0.93 | 0.056 |
| GO:0006066 | alcohol metabolic process | 0.58% | -2.5498 | 0.843 | 0.057 |

**Supplementary File 15. Top 25 Biological Process GO Annotations from REViGO for Differentially Expressed *O. amblystomatis* genes.**
