# Supplementary figures and images for: Transcriptome analysis illuminates the nature of the intracellular interaction in a vertebrate-algal symbiosis
Source: eLife. 2017 May 2;6:e22054. doi: 10.7554/eLife.22054 (PMC5413350; doi:10.7554/eLife.22054)

### REVIGO Gene Ontology treemap

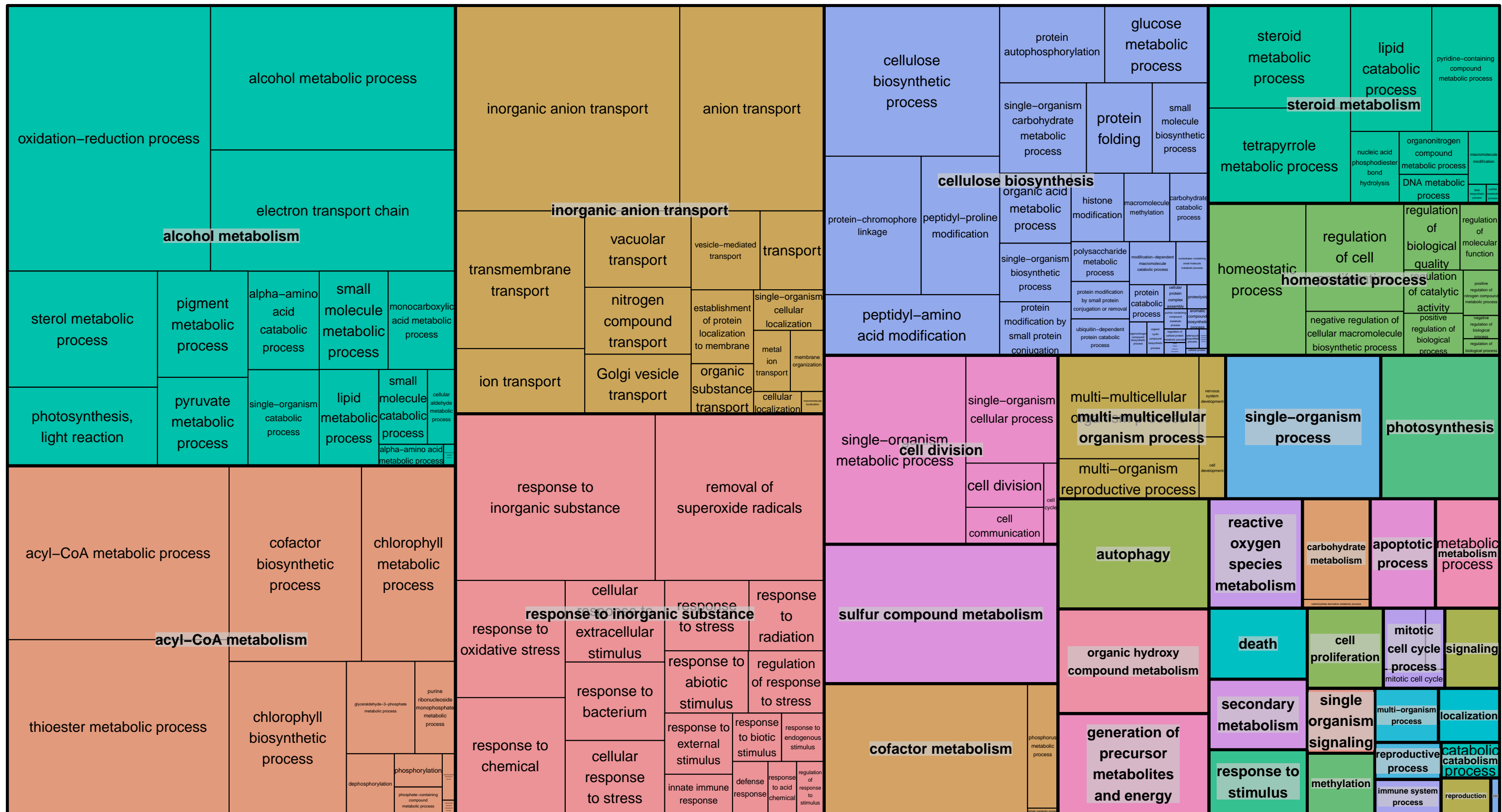

Supplement: Supplementary file 16. — DOI: http://dx.doi.org/10.7554/eLife.22054.043 [file elife-22054-supp16.pdf]

## REVIGO Gene Ontology treemap

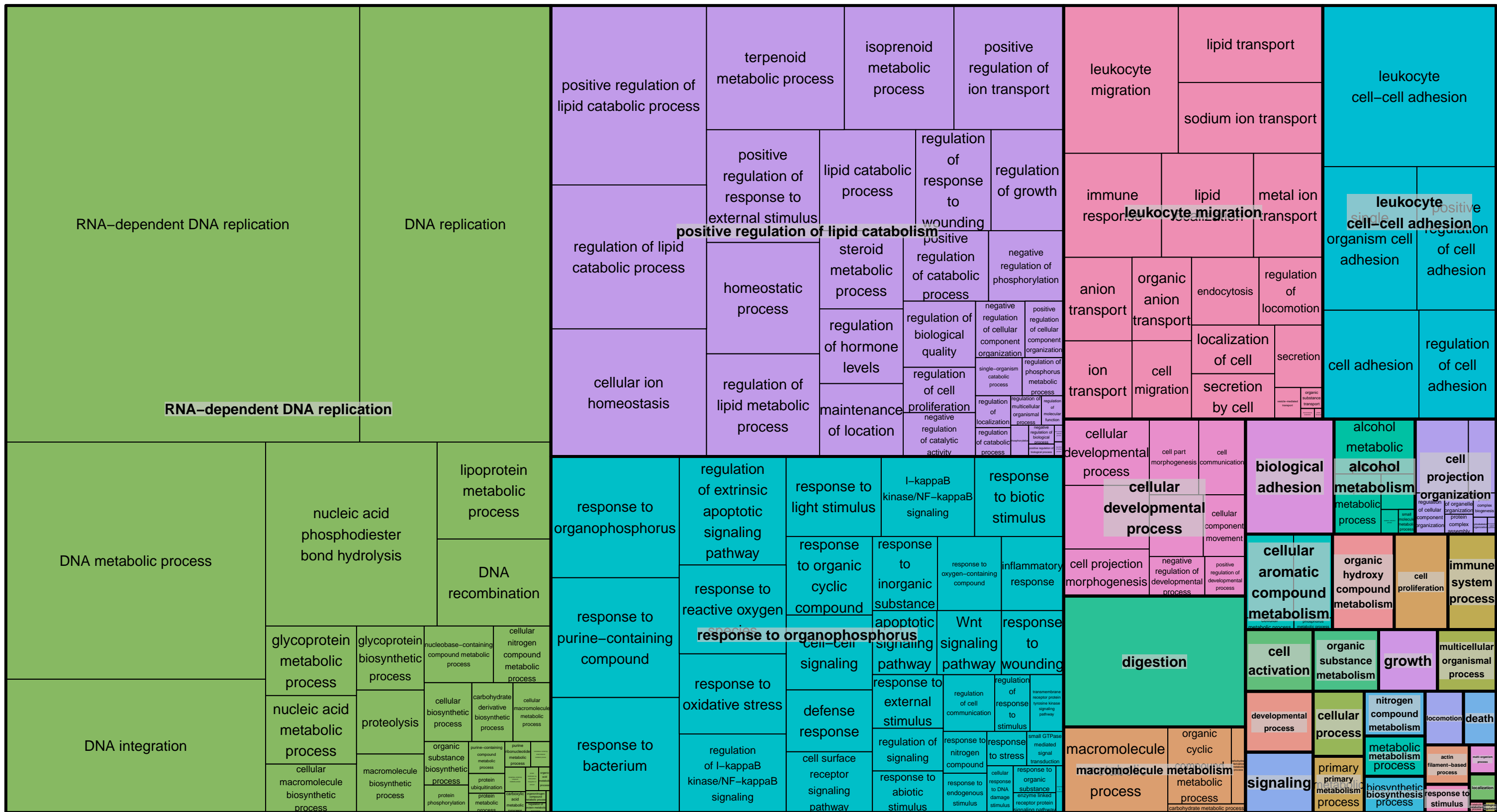

Supplement: Supplementary file 18. — DOI: http://dx.doi.org/10.7554/eLife.22054.045 [file elife-22054-supp18.pdf]
