# Supplementary material for: Transcriptome analysis illuminates the nature of the intracellular interaction in a vertebrate-algal symbiosis
Source: eLife. 2017 May 2;6:e22054. doi: 10.7554/eLife.22054 (PMC5413350; doi:10.7554/eLife.22054)
Supplement: Supplementary file 17. — DOI: http://dx.doi.org/10.7554/eLife.22054.044 [file elife-22054-supp17.docx]

| term_ID | description | frequency | log10 p-value | uniqueness | dispensability |
| --- | --- | --- | --- | --- | --- |
| GO:0006278 | RNA-dependent DNA replication | 0.45% | -21.1487 | 0.909 | 0 |
| GO:0007159 | leukocyte cell-cell adhesion | 0.00% | -3.8239 | 0.949 | 0 |
| GO:0046683 | response to organophosphorus | 0.01% | -2.1308 | 0.854 | 0 |
| GO:0022610 | biological adhesion | 1.21% | -1.394 | 0.994 | 0 |
| GO:0002376 | immune system process | 0.63% | -0.6295 | 0.994 | 0 |
| GO:0032502 | developmental process | 1.39% | -0.5622 | 0.994 | 0 |
| GO:0023052 | signaling | 3.84% | -0.5591 | 0.994 | 0 |
| GO:0040007 | growth | 0.08% | -0.4967 | 0.994 | 0 |
| GO:0032501 | multicellular organismal process | 0.79% | -0.4947 | 0.994 | 0 |
| GO:0009987 | cellular process | 65.99% | -0.4555 | 0.998 | 0 |
| GO:0008152 | metabolic process | 82.18% | -0.336 | 0.999 | 0 |
| GO:0040011 | locomotion | 1.44% | -0.2824 | 0.994 | 0 |
| GO:0050896 | response to stimulus | 8.82% | -0.1712 | 0.995 | 0 |
| GO:0051704 | multi-organism process | 2.77% | -0.1064 | 0.994 | 0 |
| GO:0051179 | localization | 17.86% | -0.1001 | 0.995 | 0 |
| GO:0065007 | biological regulation | 14.92% | -0.0185 | 0.995 | 0 |
| GO:0071704 | organic substance metabolic process | 56.18% | -0.5529 | 0.986 | 0.012 |
| GO:0009056 | catabolic process | 9.63% | -0.0234 | 0.987 | 0.024 |
| GO:0007586 | digestion | 0.01% | -3.2596 | 0.893 | 0.028 |
| GO:0050900 | leukocyte migration | 0.01% | -2.3197 | 0.823 | 0.03 |
| GO:0008283 | cell proliferation | 0.11% | -0.7029 | 0.957 | 0.036 |
| GO:0050996 | positive regulation of lipid catabolic process | 0.00% | -3.8239 | 0.752 | 0.037 |
| GO:0009058 | biosynthetic process | 30.33% | -0.3024 | 0.986 | 0.04 |
| GO:0016265 | death | 0.28% | -0.2402 | 0.954 | 0.045 |
| GO:0006807 | nitrogen compound metabolic process | 38.65% | -0.3732 | 0.986 | 0.046 |

**Supplementary File 17. Top 25 Biological Process GO Annotations from REViGO for Differentially Expressed *A. maculatum* genes.**
